# Vibrational Spectroscopy for Cocrystals Screening. A Comparative Study

**DOI:** 10.3390/molecules23123263

**Published:** 2018-12-10

**Authors:** Marisa Rodrigues, João Lopes, Mafalda Sarraguça

**Affiliations:** 1 LAQV/REQUIMTE, Departamento de Ciências Químicas, Faculdade de Farmácia, Universidade do Porto, Rua Jorge Viterbo Ferreira, 228, 4050-313 Porto, Portugal; up201305481@fc.up.pt; 2 Research Institute for Medicines (iMed.Lisboa), Faculdade de Farmácia, Universidade de Lisboa, Av. Prof. Gama Pinto, 1649-003 Lisboa, Portugal, jlopes@ff.ulisboa.pt

**Keywords:** pharmaceutical cocrystals, screening, cocrystallization by sonication, mid infrared spectroscopy, near infrared spectroscopy, Raman spectroscopy

## Abstract

A recurrent problem faced by the pharmaceutical industry when formulating drug products concerns poorly soluble drugs, which, despite having desirable pharmacological activity, present limited bioavailability. Cocrystallization is growing up as a possible approach to tackle this problem. Cocrystals are crystalline materials comprising at least two components, solid at room temperature, and held together by non-covalent bonds. The increasing interest in these compounds is steadily demanding faster, simpler, and more reliable methods for the task of screening new cocrystals. This work aims at comparing the performance of three vibrational spectroscopy techniques (mid infrared, near infrared, and Raman spectroscopy) for cocrystals screening. Presented results are based on hydrochlorothiazide, a poorly soluble drug belonging to class IV of the Biopharmaceutical Classification System. The implemented cocrystal screening procedure tested six coformers (all considered safe for human administration) added according to a drug:coformer ratio of 1:1 and 1:2 and seven solvents with different polarity. The screening method chosen was based on slurry cocrystallization performed by sonication (ultrasound assisted) in a 96-well plate. Results show that all evaluated vibrational spectroscopy techniques provided important information regarding cocrystal formation, including information on the groups involved in the cocrystallization and purity, and can be used for the screening task.

## 1. Introduction

There has been an increasing research activity on cocrystals over the last three decades. This interest in cocrystals is understandable, given that this unique crystal engineering approach has proven to be advantageous, mainly for the enhancement of active pharmaceutical ingredients (API’s) solubility profiles, which directly affects drug dissolution and bioavailability [1]. The design of cocrystals may be achieved by a combination of theoretical and/or experimental strategies. A multifactorial rational approach must be initiated by complete and exhaustive characterization of the API followed by coformer selection and cocrystallization method selection. The selection of the correct coformer can be done applying crystal engineering principles by understanding the intermolecular interactions, which could be used in the design and modulation of new solid forms with fine-tuned properties [2]. A rational synthesis of cocrystals can be achieved by combining the knowledge of geometric analysis [3] and by using the so-called “hydrogen-bond rules” [4]. Supramolecular synthons recognition is based on the determination of specific intermolecular interactions such as hydrogen or halogen bonds and stacking interactions, among others. This approach can be very efficient for relatively strong interactions, but comparison of strengths between different types of specific intermolecular interactions (e.g., weak hydrogen bonds or van der Waals forces) can be almost impossible due to the dependence of the energy of interactions on interatomic distances in different types of intermolecular interactions. In addition, in the case of big molecules, the contribution of general dispersion and electrostatic interactions to the total energy of interaction may be comparable with the energy of specific interactions. Theoretical approaches, such as the Basic Structural Motifs (BSM) [5] and the evaluation of the Hansen Solubility Parameters (HSP), can be used to solve some of the aforementioned problems [6]. From another perspective, statistical models can be developed using the Cambridge Structural Database (CSD) [7]. These methods can be used individually, or in combination, as strategies to cocrystals design and coformer selection. However, all of them are based on complex methodologies and are time-consuming. Due to a large number of viable coformers for a single API, a fast method for cocrystal screening is of upmost importance in the development phase [8]. Some examples on cocrystal screening involve mechanochemical grinding [9,10], solvent, and solvent drop sonication in 96-well plates [8,11,12]. Development of miniaturized systems based on microfluidics for cocrystal screening has been increasing [13,14,15].

In this work, a screening procedure based on slurry cocrystallization by sonication in a 96-well plate is performed [12]. Hydrochlorothiazide (HTZ) was the selected API. It is poorly soluble, belonging to class IV of the Biopharmaceutical Classification System (BCS), and therefore a good candidate for cocrystal formation [16,17,18,19,20,21,22]. Six coformers, all considered safe for consumption, in the drug:coformer ratio of (1:1) and (1:2) and seven solvents with different polarity were tested in a total of 84 experiments. To the best of our knowledge, none of these coformers have been used until now to produce cocrystals with HTZ. 

The main objective of the work is to analyze the screening cocrystallization products with three vibrational spectroscopic techniques, mid infrared spectroscopy (MIRS), near infrared spectroscopy (NIRS), and Raman spectroscopy (RS), in order to compare them in three important features for cocrystals screening. 1) To provide information on the formation of the cocrystal; 2) to assess the groups involved in the cocrystallization, and 3) to infer on their purity. Vibrational spectroscopy methods have several advantages for cocrystal screening. These methods are fast, non-destructive, can be coupled to a microscope and provide simultaneously chemical and physical information [23,24]. Several studies have shown their ability for crystal/cocrystal screening [25,26,27,28,29,30,31,32]. Additionally, they can also be very useful for determining the purity of the final product when coupled with chemometric methods such as principal component analysis (PCA) [27]. Nevertheless, a direct comparison between the three techniques for cocrystal screening as never been done before. 

## 2. Results and Discussion

The cocrystallization products were analyzed with MIRS, NIRS, and RS. Cocrystal formation was assessed through a visual comparison between the spectrum of the cocrystallization products with the physical mixture (PM). This process was repeated for all potential coformers. Additionally, spectra were analyzed by principal component analysis (PCA) to evaluate the similarity between cocrystallization products obtained with different solvents and PM and in this way their purity. 

After the completion of the cocrystal screening procedure, it was verified that the wells containing ascorbic acid showed an apparently degraded product. Therefore, the results from these systems were not considered. The analysis of the systems containing adenine showed that there were no differences between the spectra of the cocrystallization products and the PM. All vibrational spectroscopy techniques were consistent. It can be concluded that no cocrystal was obtained between HTZ and adenine. The results for the remaining systems will be analyzed separately.

### 2.1. Hydrochlorathiazide:Tromethamine (HTZ:Tris)

**Figure 1:**
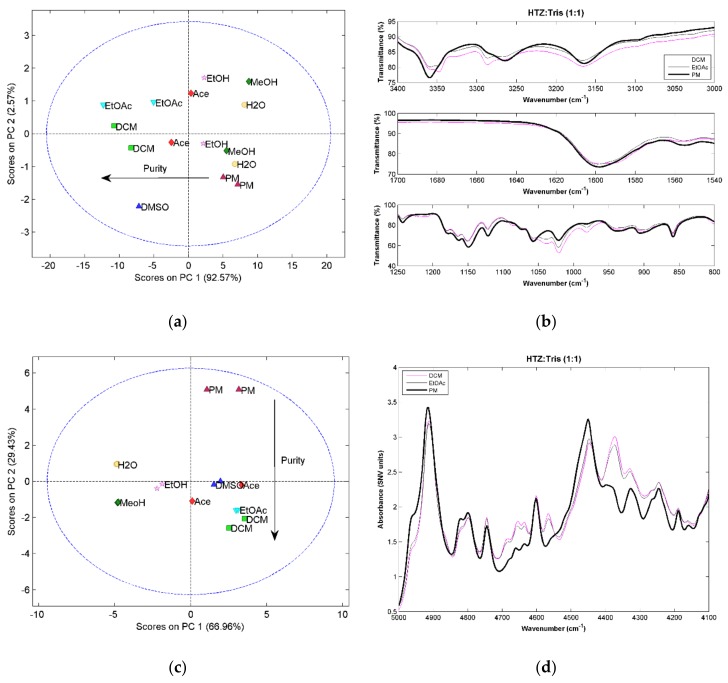

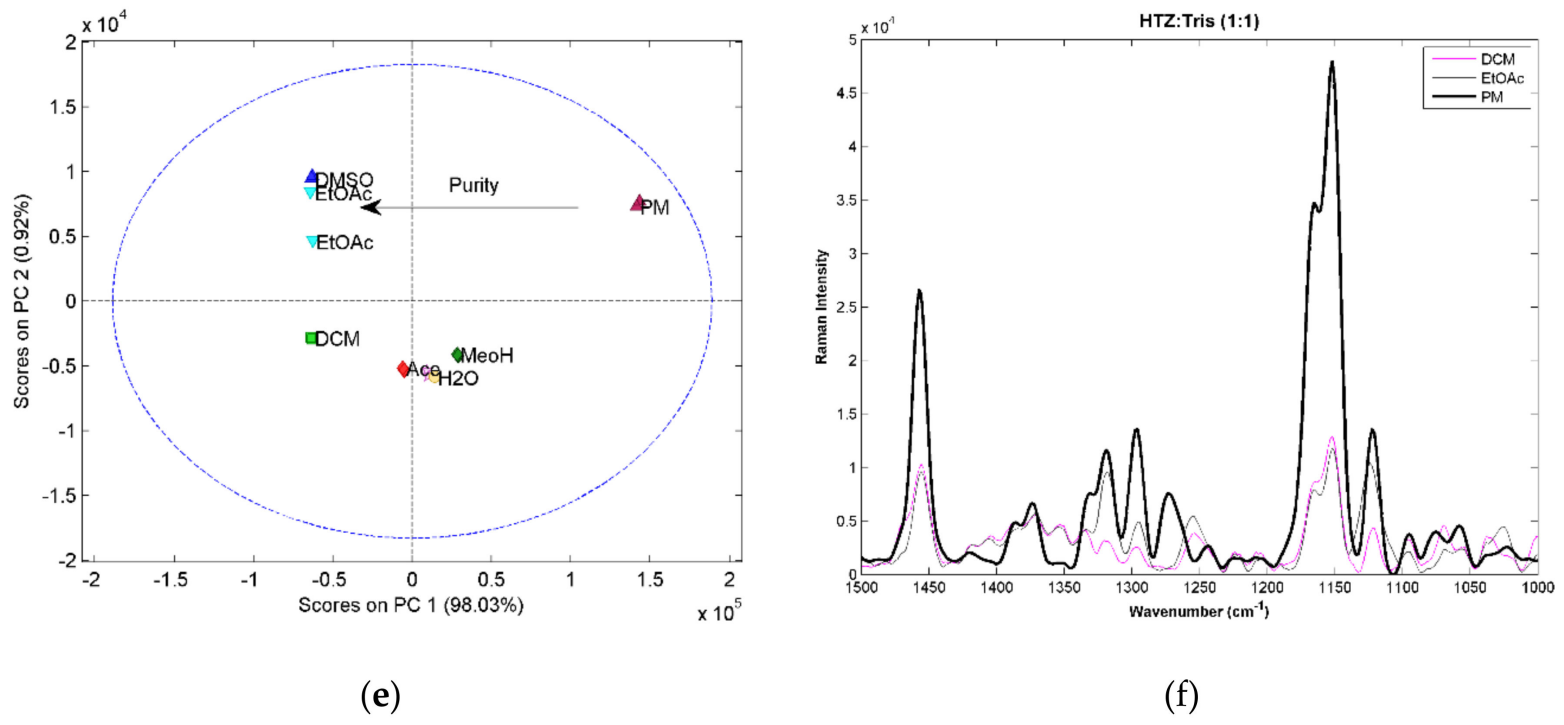
(**a**) Principal component analysis (PCA) score plot constructed with the mid infrared spectroscopy (MIRS) spectra from the system HTZ:Tris (1:1); (**b**) MIRS spectra of the samples produced with DCM and EtOAc and from the physical mixture (PM); (**c**) PCA score plot constructed with the near infrared spectroscopy (NIRS) spectra from the system HTZ:Tris (1:1); (**d**) NIRS spectra of the samples produced with DCM and EtOAc and from the PM; (**e**) PCA score plot constructed with the Raman spectroscopy (RS) spectra from the system HTZ:Tris (1:1); (**f**) RS spectra of the samples produced with DCM and EtOAc and from the PM. Colors refer to different solvents (dark green: MeOH; pink: EtOH; red: Ace; green: DCM; dark blue: DMSO; blue: EtOAc; yellow: H_2_O; dark red: PM).

The score plot of the first two components from the PCA model constructed with the vibrational spectra for the HTZ:Tris (1:1) systems can be seen in Figure 1. The MIRS results (Figure 1a) a show that the samples more distant to the PM in the first component are the samples produced with DCM and EtOAc, meaning that these samples are the ones that present more differences in the spectra when compared with the PM. The visual analysis of the spectra (Figure 1b) revealed that the MIRS show differences consistent with cocrystal formation. The score plot for NIRS (Figure 1c) shows a similar trend in the second component. In the case of NIRS, the first component appears to reflect differences related with physical properties (e.g., particle size). Nevertheless, the samples obtained with DCM and EtOAc were also the ones that presented a higher degree of differences when comparing their spectra with the spectrum of the PM (Figure 1d). RS confirms the above results. Systems yielding a more promising cocrystal were obtained with DCM and EtOAc (Figure 1e,f). The spectra of the 1:1 ratio systems were compared with the spectra of the 1:2 ratio systems and it was verified that the samples obtained with the two ratios show similar spectra. In fact, it can be seen that the changes in the spectra are the same. Nevertheless, the 1:2 ratio systems seem to present more differences in relation to the PM. Therefore, it can be inferred that the 1:2 ratio systems formed a large amount of cocrystal. 

**Figure 2:**
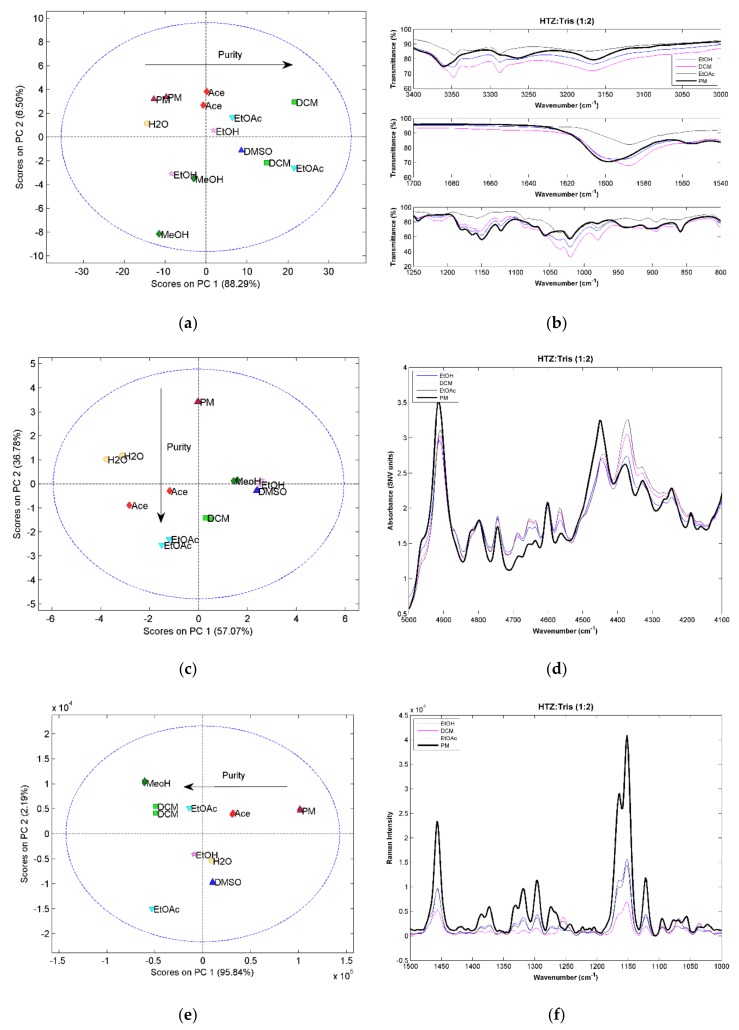
(**a**) PCA score plot constructed with the MIRS spectra from the system HTZ:Tris (1:2); (**b**) MIRS spectra of the samples produced with EtOH, DCM, and EtOAc and from the PM; (**c**) PCA score plot constructed with the NIRS spectra from the system HTZ:Tris (1:2); (**d**) NIRS spectra of the samples produced with EtOH, DCM, and EtOAc and from the PM; (**e**) PCA score plot constructed with the RS spectra from the system HTZ:Tris (1:2); (**f**) RS spectra of the samples produced with EtOH, DCM, and EtOAc and from the PM. Colors refer to different solvents (dark green: MeOH; pink: EtOH; red: Ace; green: DCM; dark blue: DMSO; blue: EtOAc; yellow: H_2_O; dark red: PM).

For the 1:2 ratio systems, the score plot of the PCA constructed with the MIRS spectra (Figure 2a) shows a trend in the first component associated with the purity of the samples. Samples further away from the PM in the first component are the ones with a more pure cocrystal. This conclusion was supported by the analysis of the spectra. Differences in the purity of the samples can be seen in the example given in Figure 2b. The samples produced with DCM and EtOAc, the ones that are farther away in PC1 from the PM, present spectra with significant differences compared with the PM. The main differences can be seen in three regions. In the amine region between 3400 cm^−1^ and 3000 cm^−1^, there are shifts in two bands corresponding to amine groups of HTZ and Tris. In the second region, around 1600 cm^−1^, the band corresponding to the vibration of the heterocyclic ring system of HTZ and to the vibration of the amine group in Tris shifted from 1600 cm^−1^ to 1585 cm^−1^. The third region between 1200 cm^−1^ and 800 cm^−1^ presents several differences associated with the vibration of the S=O groups of HTZ. The spectra from the sample produced with EtOH, which in the PCA score plot is in between the PM and the EtOAc samples, have bands characteristic of the PM and cocrystal (for example, the bands at 3350 cm^−1^ and 1600 cm^−1^), indicating that the sample is impure. The analysis of the NIRS spectra by PCA confirms that a cocrystal was in fact produced. However, the samples are not pure (Figure 2c). Again, the differences are seen in the second component. The analysis of the spectra (Figure 2d) show differences when compared with the PM, mainly in the region between 5000 cm^−1^ and 4100 cm^−1^. This spectral region encloses the first overtone region of the amine group. The PCA score plot obtained with the RS spectra shows differences in the purity of the cocrystal reflected on the first component (Figure 2e). The region of the symmetric and asymmetric stretch of the S=O group of HTZ (between 1400 cm^−1^ and 1300 cm^−1^) is the one with a large number of differences associated with cocrystal formation (Figure 2f). 

The three techniques showed the formation of a cocrystal between HTZ and Tris. The PCA applied to the spectra also revealed that the extension of the cocrystal formation was dependent of the solvent used. In this case, the samples produced with DCM and EtOAc were the ones that produced a more pure cocrystal. The groups involved in the formation of the cocrystal are the sulfonamide group of HTZ and the amine group of Tris.

### 2.2. Hydrochlorothiazide:Malic Acid (HTZ:Mal)

The three vibrational spectroscopic techniques were coherent regarding the HTZ:Mal (1:1) systems. Samples processed with DCM, Ace, EtOAc, and DMSO produced cocrystals. However, the spectral analysis indicated incomplete cocrystal formation (results not shown). The comparison between the 1:1 ratio and 1:2 ratio systems showed that despite similarity between spectra, the 1:2 ratio evidenced a superior cocrystal yield (results not shown). Therefore, a more detailed analysis will be presented for the 1:2 ratio systems. 

**Figure 3:**
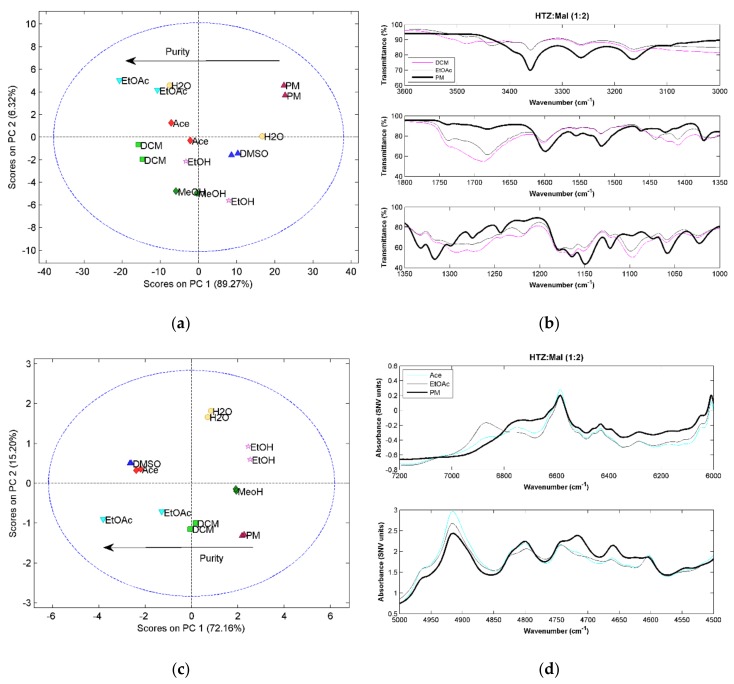

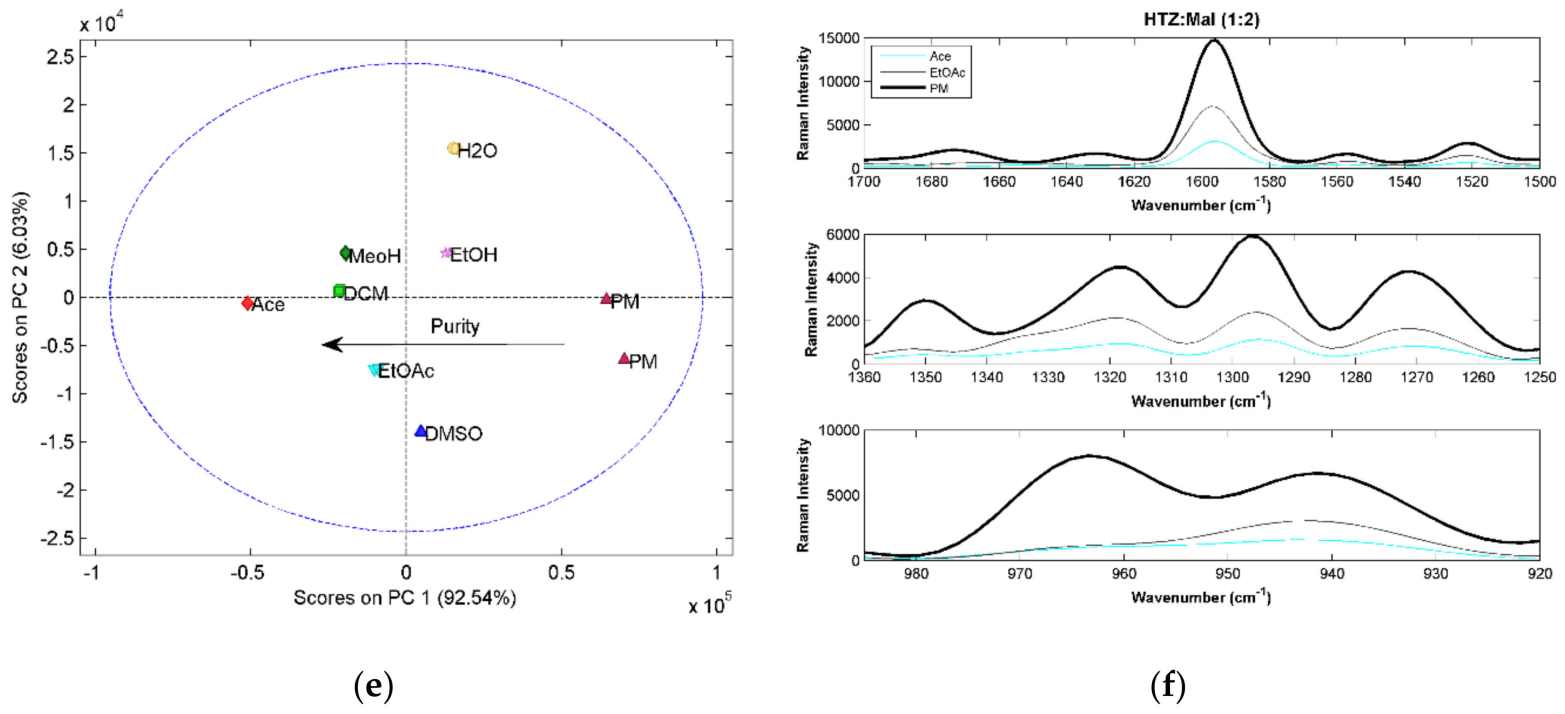
(**a**) PCA score plot constructed with the MIRS spectra from the system HTZ:Mal (1:2); (**b**) MIRS spectra of the samples produced with DCM and EtOAc and from the PM; (**c**) PCA score plot constructed with the NIRS spectra from the HTZ:Mal (1:2); (**d**) NIRS spectra of the samples produced with Ace and EtOAc and from the PM; (**e**) PCA score plot constructed with the RS spectra from the HTZ:Mal (1:2); (**f**) RS spectra of the samples produced with Ace and EtOAc and from the PM. Colors refer to different solvents (dark green: MeOH; pink: EtOH; red: Ace; green: DCM; dark blue: DMSO; blue: EtOAc; yellow: H_2_O; dark red: PM).

All HTZ:Mal (1:2) experiments produced a cocrystal. This can be confirmed by the score plots for the three techniques showing a similar pattern. The first principal component seems to be related with cocrystal purity. The MRIS results (Figure 3a) show that the samples presenting superior differences in relation to the PM were produced with EtOAc and DCM. The NIRS (Figure 3c) and RS (Figure 3e) results seem to confirm this result. 

Comparing the spectra obtained from the cocrystallization systems and the PM, the MIRS spectra is, again, the technique providing the most detailed information regarding the groups involved in the cocrystal formation (Figure 3b). Several new bands and band shifts can be observed. The differences verified between 3500 cm^−1^ and 3400 cm^−1^ are related to the stretching vibration of the OH group from acid groups of malic acid. The carbonyl group stretching vibrations, in the region of 1700 cm^−1^ also show differences in the cocrystal spectra. Emerging bands and band shifts are seen in the region of the asymmetric and symmetric stretching of the S=O groups of HTZ (between 1350 cm^−1^ and 1100 cm^−1^). The main differences between the cocrystal and PM samples in the NIRS region (Figure 3d) are seen on the second overtone of the vibration of the alcohol group from acid malic (~6900 cm^−1^) and the region between 4600 cm^−1^ and 4550 cm^−1^ on the combination band region of the alcohol, amine, and amide groups. On the RS spectra (Figure 3f) the bands corresponding to C=O vibrations at 1635 cm^−1^ and 1675 cm^−1^ from malic acid and to the S=O vibrations around 1300 cm^−1^ and SN stretching plus NH deformation of HTZ between 980 cm^−1^ and 920 cm^−1^ are the major differences between the cocrystal and the PM spectrum. 

The cocrystal formation was detected by the three techniques, as well as differences in purity. The NIRS method provides less information regarding the groups involved in the cocrystal formation. The sulfonamide group of HTZ and the acid group of malic acid were identified as the groups involved in the formation of the cocrystal. 

### 2.3. Hydrochlorothiazide:Citric Acid (HTZ:Cit)

**Figure 4:**
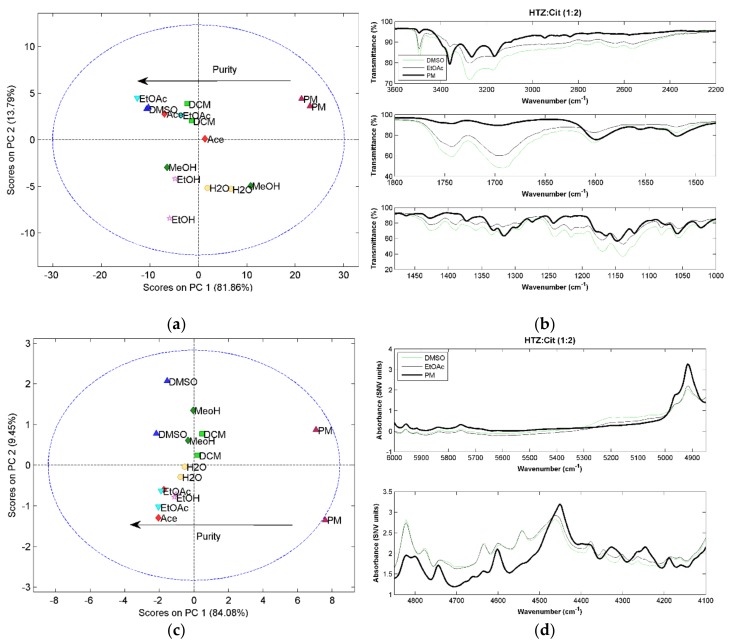

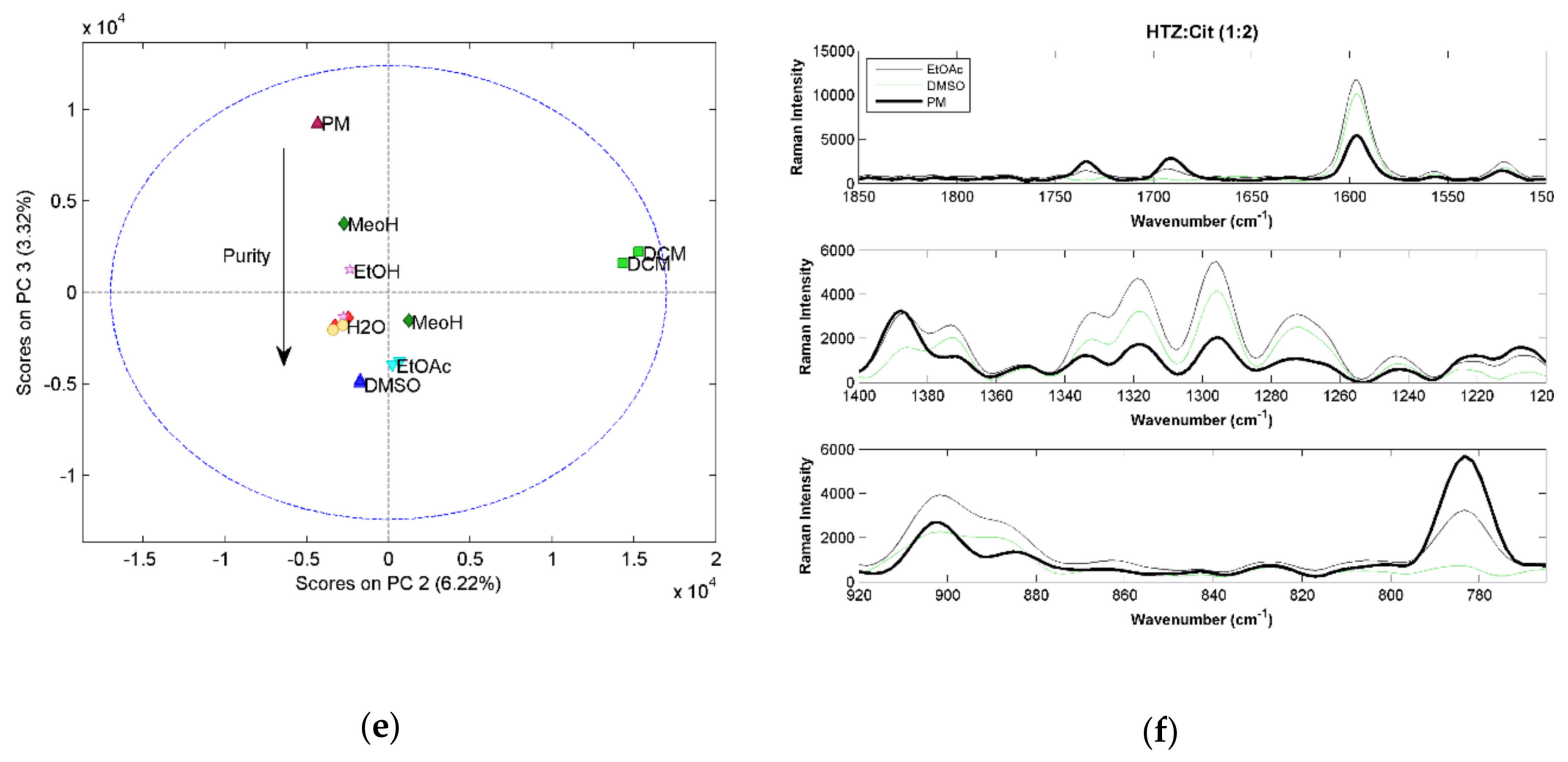
(**a**) PCA score plot constructed with the MIRS spectra from the system HTZ:Cit (1:2); (**b**) MIRS spectra of the samples produced with DMSO and EtOAc and from the PM; (**c**) PCA score plot constructed with the NIRS spectra from the HTZ:Cit (1:2); (**d**) NIRS spectra of the samples produced with DMSO and EtOAc and from the PM; (**e**) PCA score plot constructed with the RS spectra from the HTZ:Cit (1:2); (**f**) RS spectra of the samples produced with DMSO and EtOAc and from the PM. Colors refer to different solvents (dark green: MeOH; pink: EtOH; red: Ace; green: DCM; dark blue: DMSO; blue: EtOAc; yellow: H_2_O; dark red: PM).

For all HTZ:Cit (1:1) systems, a low purity cocrystal was obtained, as in the case of malic acid and tromethamine (results not shown). For the HTZ:Cit (1:2) systems, it is clear after the PCA that the PM spectrum presents significant differences to all spectra of the cocrystallization products, indicating cocrystal formation (Figure 4a,c,e). For RS, the difference is perceived in the score plot of the second against the third component (Figure 4e). Additionally, there is a visible separation between the samples produced with DCM and other solvents. A more detailed analysis of the RS spectra of these samples show incongruences in the spectra when compared with the other cocrystallization products. Since none of the other two techniques showed this trend, it was considered that these two spectra were outliers. 

The PCA results show that the cocrystals produced with DMSO and EtOAc are the purest. The MIRS spectra of these samples (Figure 4b) presents alterations in the HTZ bands between 3300 cm^−1^ and 3100 cm^−1^ and between 1400 cm^−1^ and 1100 cm^−1^ associated with the NH_2_ and S=O groups, respectively. The region associated with the C=O group vibration from citric acid (~1700 cm^−1^ and 1750 cm^−1^) clearly changed. In the near infrared region, the differences between the PM and cocrystal spectra are in the region of the first and second overtone region of the OH group vibration of citric acid (~5200 cm^−1^ and ~4700 cm^−1^) and in the first overtone of the amine group of HTZ (~4550 cm^−1^) (Figure 4d). The changes on the RS spectra (Figure 4f) are related with the C=O group of citric acid and the S=O group of HTZ.

As in the previous systems, the vibrational spectroscopic techniques were able to detect the cocrystal formation and differences in their purity. The groups involved in the cocrystallization are the acid group of the citric acid and the sulfonamide group of HTZ.

### 2.4. Hydrochlorothiazide:d-Mannitol (HTZ:Man)

**Figure 5:**
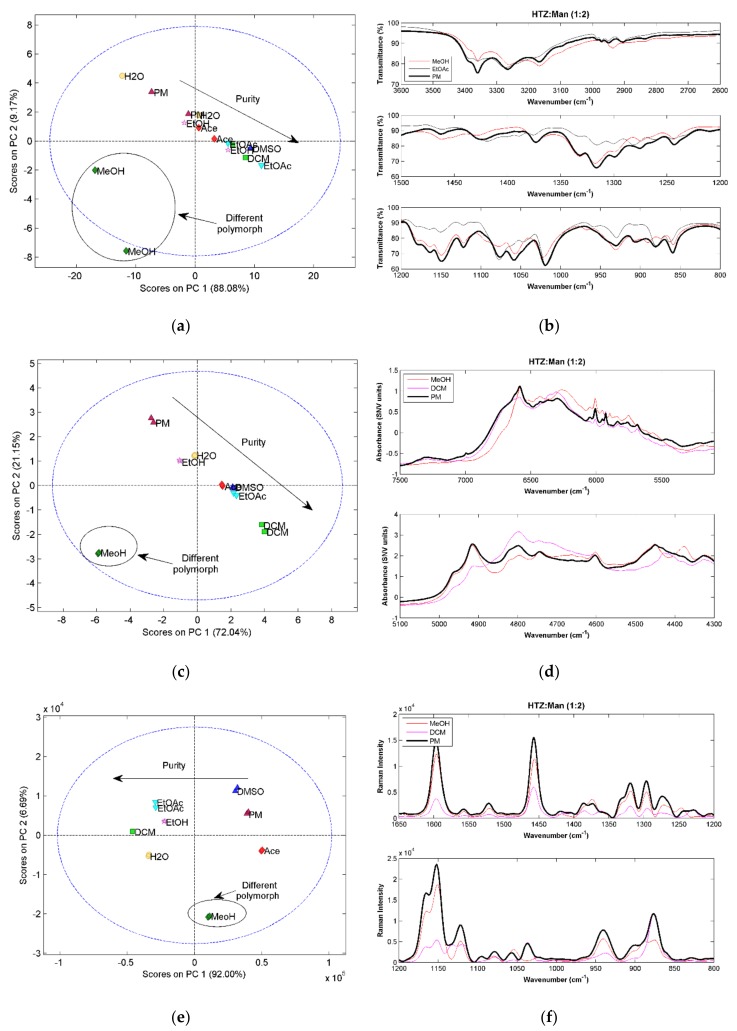
(**a**) PCA score plot constructed with the MIRS spectra from the system HTZ:Man (1:2); (**b**) MIRS spectra of the samples produced with, MeOH and EtOAc and from the PM; (**c**) PCA score plot constructed with the NIRS spectra from the HTZ:Man (1:2); (**d**) NIRS spectra of the samples produced with MeOH and DCM and from the PM; (**e**) PCA score plot constructed with the RS spectra from the HTZ:Man (1:2); (**f**) RS spectra of the samples produced with MeOH and DCM and from the PM. Colors refer to different solvents (dark green: MeOH; pink: EtOH; red: Ace; green: DCM; dark blue: DMSO; blue: EtOAc; yellow: H_2_O; dark red: PM).

As for the previously analyzed systems, in this case, the 1:1 ratio also gave cocrystals with lower purity. Therefore, only the results for the 1:2 ratio systems will be discussed. Figure 5a,c,e shows the score plots of the PCA model constructed with spectra of the HTZ:Man (1:2) systems. All the techniques display, in the first component, the same trend. The samples obtained with EtOAc (in the case of MIRS) and DCM (in the other two cases) show the major differences in relation to the PM, indicating the possibility of cocrystal formation. Additionally, in the second component, the samples produced with MeOH are separated from the remaining samples, also indicating that a cocrystal was obtained, but with a spectral signature different from the one of the samples produced with the other solvents. The detailed analysis of the spectra Figure 5b,d and Figure 5,f shows that although the spectra of the samples produced with MeOH and EtOAc show differences, the same groups appear to be involved in the cocrystal formations. These groups are the alcohol group of d-mannitol and the S=O and amine groups of HTZ. Additionally, it is clear that the cocrystals are not pure, even the ones obtained with EtOAc and DCM (first component). 

The existence of two different cocrystal polymorphs can be a possible explanation for the observed differences. Polymorphism in cocrystals is very well documented [32,33], therefore, it is possible that differences in the solvent polarity will origin different polymorphs of the same cocrystal.

The results for this system show that vibrational spectroscopy can be very helpful, not only in determining the formation and purity of the cocrystal, but also in the recognition of different polymorphs.

## 3. Materials and Methods

HTZ (>98% purity), was acquired from Alfa Aesar (Karlsruhe, Germany), adenine (Ade) (>99.0% purity), citric acid monohydrated (Cit) (>99.0% purity), ascorbic acid (Asc) (>99.0% purity), and tromethamine (Tris) (>99.0% purity) were acquired from Sigma-Aldrich (Darmstadt, Germany), acid malic (Mal) (>99.5% purity) was acquired from Merck (Darmstadt, Germany) and d-Mannitol (Man) (>99.0% purity) was acquired from Riedel-de-Haën (Seelze, Germany). Methanol (MeOH) and acetone (Ace) were acquired form VWR Chemicals (Le Périgares, France), dichloromethane (DCM) and ethyl acetate (EtOAc) were acquired from Sigma-Aldrich (Darmstadt, Germany), ethanol (EtOH) was acquired from Chem-Lab NV (Zedelgem, Belgium), and dimethyl sulfoxide (DMSO) from Riedel-de-Haën (Seelze, Germany). All solvents were analytical grade.

### 3.1. Cocrystallization Experiments

**Figure 6:**
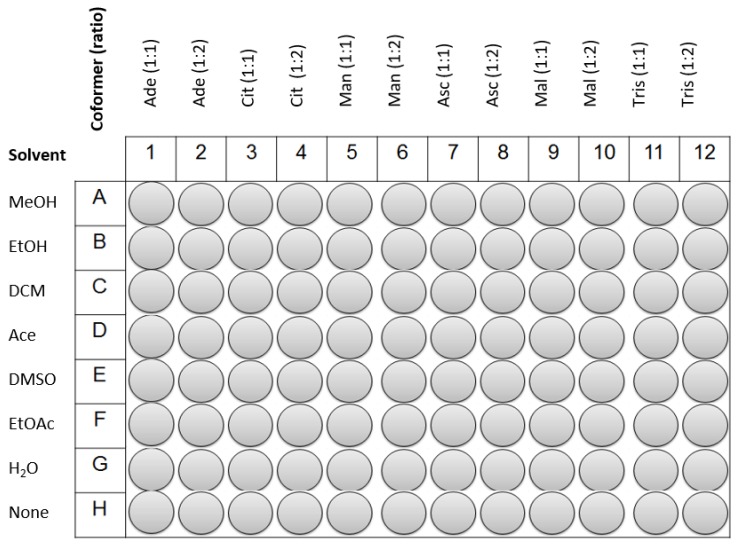
Coformers, ratio of drug:coformer and solvents used on the 96-well plate. The H line refers to the respective physical mixture (PM).

HTZ and the coformers were weighted and transferred to a 96-well plate of 300 µL. The quantity of HTZ dispended was 25 mg (0.084 mmol) in each well. The coformers were added posteriorly in the pre-defined ratio. Reference samples and physical mixtures (PM) were placed on the last line of the plate (see Figure 6). An amount of 30 µL of each solvent was added to the wells, except in the wells containing the PM. In the end, the 96-well plate was sealed with parafilm and subjected to sonication in an ultrasound bath for 2 h. Afterwards, the plate was unsealed and solvents were left to evaporate in a fume hood during a week at room temperature. After this period, the content of each well was analyzed by MIRS, NIRS, and RS.

### 3.2. Mid Infrared Spectroscopy

A Fourier transform mid infrared spectrometer (Frontier, PerkinElmer, Beaconsfield, UK) equipped with an attenuated total reflectance (ATR) accessory (PerkinElmer, Beaconsfield, UK) was used to acquire mid infrared spectra between 4000 cm^−1^ and 600 cm^−1^. The resolution was set to 4 cm^−1^ and each stored spectrum is the average of 32 co-added scans. The spectrophotometer has a deuterated triglycine sulphate (DTGS) detector and a mid-infrared light source. To allow optimal contact between the sample and the crystal as well as sample-to-sample reproducibility, the ATR accessory is equipped with a pressure arm with indication of force. Samples were directly applied on the ATR crystal and the same force was applied in each measurement. The instrument is controlled via the Spectrum software (PerkinElmer, Beaconsfield, UK). A background was made with the ATR accessory empty. For each sample, two replicates were taken.

### 3.3. Near Infrared Spectroscopy

Near infrared spectra in the range 10,000 to 4000 cm^−1^ were acquired by a Fourier transform NIR analyzer (FTLA2000, ABB, Québec, Canada). The spectrophotometer is equipped with an indium-gallium-arsenide (InGaAs) detector and powder sampling accessory (ACC101, ABB, Québec, Canada) with a 2 cm diameter window. The resolution was set to 2 cm^−1^ and each stored spectrum was an average of 64 co-added scans. The instrument is controlled via the Grams LT software (version 7, ABB, Québec, Canada). The background was taken with a certified material (polytetrafluoroethylene, PTFE, SKG8613G, ABB, Québec, Canada). For each sample, two spectral replicates were made.

### 3.4. Raman Spectroscopy

Raman spectra were acquired using a Raman CORA5700 (Cora 5X000 Raman spectrometer, Anton Paar, Ashland, VA, USA) equipped with a 785 nm laser. Spectra were collected between 98 and 1800 cm^−1^ setting the laser power to 450 mW. Sample exposure time was 20 s. For each sample, two spectral replicates were made by placing the powder in a glass support that was placed inside the spectrometer aligned with the laser. 

### 3.5. Data Analysis

Principal component analysis (PCA) [34] was used to analyze the spectra in order to compare the spectrum of the PM with the spectrum of the products obtained with each solvent. In this way, it can be infer the effect of the solvents in the formation and purity of the cocrystals. 

Before PCA, MIRS, and NIRS spectra were pre-processed with standard normal variate (SNV) to correct for disturbances caused by light scattering (e.g., resulting from variable particle size). Data were then mean centred immediately before the estimation of the PCA models. Matlab version 8.3 (MathWorks, Natick, MA, USA) and the PLS Toolbox version 7.5 (Eigenvector Research Inc., Wenatchee, WA, USA) were used for this analysis.

## 4. Conclusions

All vibrational spectroscopic techniques prove to be able to detect cocrystal formation. With PCA, it was possible to infer about the purity of the different cocrystallization products, and how the solvent affected this property. In the case of D-mannitol, two different polymorphs were also detected. NIRS does not provide information about the vibration of the S=O groups of HTZ. This fact can be a disadvantage of the technique, in this particular case, since this group was involved in the cocrystal formation in all cases. Regarding Raman spectroscopy, and because the used spectrometer did not span the region between 4000 cm^−1^ and 1850 cm^−1^ (a region with information on the amine and alcohol groups), we conclude that it is less informative when compared with MIRS. 

Among the coformers used in this work (with the exception of ascorbic acid that was not analyzed), adenine was the only not forming a cocrystal with hydrochlorothiazide. In all remaining cases, it was demonstrated that the ratio 1:1 is able to form the cocrystal but with a lower purity than the 1:2 ratio. Hydrochlorothiazide seems to have a preference to form 1:2 (drug:coformer) ratio cocrystals, possibly due to the presence of two sulfonamide groups capable of hydrogen bonding with the coformers. However, it is not possible to claim that the cocrystal ratio was 1:2 since in the screening procedure the compounds were not dissolved, and therefore the ratio may not be the one present in the well. To determine the ratio, new experiments should be done (e.g., high performance liquid chromatography, X-ray powder diffraction). It was also shown that the solvent plays an important role on cocrystal purity. This was clearly observed from the PCA results. The solvents that produced cocrystals with higher purity were DCM and EtOAc. This may indicate a preference for less polar solvents.

Globally, this work showed that, with the equipment used, the three tested vibrational spectroscopy techniques can be useful to analyze the outcome of a cocrystal screening procedure, allowing one to confirm the cocrystal formation, purity, and eventual presence of polymorphs. These methods allow this analysis in a very simple and rapid manner, which is extremely important for cocrystal screening. 

## References

[1] Duggirala N.K., Perry M.L., Almarsson O., Zaworotko M.J. (2016). Pharmaceutical cocrystals: Along the path to improved medicines. Chem. Commun..

[2] Nangia A., Desiraju G.R., Weber E., Aoyama Y., Caira M.R., Desiraju G.R., Glusker J.P., Hamilton A.D., Meléndez A.D., Nangia A. (1998). Supramolecular Synthons and Pattern Recognition. Design of Organic Solids.

[3] Etter M.C. (1990). Encoding and Decoding Hydrogen-Bond Patterns of Organic-Compounds. Accounts Chem. Res..

[4] Jones W., Motherwell S., Trask A.V. (2006). Pharmaceutical cocrystals: An emerging approach to physical property enhancement. MRS Bull..

[5] Shishkin O.V., Zubatyuk R.I., Shishkina S.V., Dyakonenko V.V., Medviediev V.V. (2014). Role of supramolecular synthons in the formation of the supramolecular architecture of molecular crystals revisited from an energetic viewpoint. Phys. Chem. Chem. Phys..

[6] Fukte S.R., Wagh M.P., Rawat S. (2014). Coformer selection: An important tool in cocrystal formation. Int. J. Pharm. Pharm. Sci..

[7] Abramov Y.A., Loschen C., Klamt A. (2012). Rational coformer or solvent selection for pharmaceutical cocrystallization or desolvation. J. Pharm. Sci..

[8] Luu V., Jona J., Stanton M.K., Peterson M.L., Morrison H.G., Nagapudi K., Tan H. (2013). High-throughput 96-well solvent mediated sonic blending synthesis and on-plate solid/solution stability characterization of pharmaceutical cocrystals. Int. J. Pharm..

[9] Bysouth S.R., Bis J.A., Igo D. (2011). Cocrystallization via planetary milling: Enhancing throughput of solid-state screening methods. Int. J. Pharm..

[10] Lin H.L., Zhang G.C., Hsu P.C., Lin S.Y. (2013). A portable fiber-optic Raman analyzer for fast real-time screening and identifying cocrystal formation of drug-coformer via grinding process. Microchem. J..

[11] Kojima T., Tsutsumi S., Yamamoto K., Ikeda Y., Moriwaki T. (2010). High-throughput cocrystal slurry screening by use of in situ Raman microscopy and multi-well plate. Int. J. Pharm..

[12] Leung D.H., Lohani S., Ball R.G., Canfield N., Wang Y.L., Rhodes T., Bak A. (2012). Two Novel Pharmaceutical Cocrystals of a Development Compound–Screening, Scale-up, and Characterization. Cryst. Growth Des..

[13] Goyal S., Thorson M.R., Zhang G.G.Z., Gong Y.C., Kenis P.J.A. (2012). Microfluidic Approach to Cocrystal Screening of Pharmaceutical Parent Compounds. Cryst. Growth Des..

[14] Simone E., McVeigh J., Reis N.M., Nagy Z.K. (2018). A high-throughput multi-microfluidic crystal generator (MMicroCryGen) platform for facile screening of polymorphism and crystal morphology for pharmaceutical compounds. Lab Chip.

[15] Shi H.H., Xiao Y., Ferguson S., Huang X., Wang N., Hao H.X. (2017). Progress of crystallization in microfluidic devices. Lab Chip.

[16] Silva A.F.T., Sarraguca M.C., Ribeiro P.R., Santos A.O., De Beer T., Lopes J.A. (2017). Statistical process control of cocrystallization processes: A comparison between OPLS and PLS. Int. J. Pharm..

[17] Ranjan S., Devarapalli R., Kundu S., Vangala V.R., Ghosh A., Reddy C.M. (2017). Three new hydrochlorothiazide cocrystals: Structural analyses and solubility studies. J. Mol. Struct..

[18] Gopi S.P., Banik M., Desiraju G.R. (2017). New Cocrystals of Hydrochlorothiazide: Optimizing Solubility and Membrane Diffusivity. Cryst. Growth Des..

[19] Arafa M.F., El-Gizawy S.A., Osman M.A., El Maghraby G.M. (2016). Sucralose as co-crystal co-former for hydrochlorothiazide: Development of oral disintegrating tablets. Drug Dev. Ind. Pharm..

[20] El-Gizawy S.A., Osman M.A., Arafa M.F., El Maghraby G.M. (2015). Aerosil as a novel co-crystal co-former for improving the dissolution rate of hydrochlorothiazide. Int. J. Pharm..

[21] Sanphui P., Devi V.K., Clara D., Malviya N., Ganguly S., Desiraju G.R. (2015). Cocrystals of Hydrochlorothiazide: Solubility and Diffusion/Permeability Enhancements through Drug-Coformer Interactions. Mol. Pharm..

[22] Sanphui P., Rajput L. (2014). Tuning solubility and stability of hydrochlorothiazide co-crystals. Acta Crystallogr. Sect. B-Struct. Sci.Cryst. Eng. Mat..

[23] Sarraguça M.C., Lopes J.A. (2009). Quality control of pharmaceuticals with NIR: From lab to process line. Vib. Spectrosc..

[24] De Beer T., Burggraeve A., Fonteyne M., Saerens L., Remon J.P., Vervaet C. (2011). Near infrared and Raman spectroscopy for the in-process monitoring of pharmaceutical production processes. Int. J. Pharm..

[25] Mukherjee A., Tothadi S., Chakraborty S., Ganguly S., Desiraju G.R. (2013). Synthon identification in co-crystals and polymorphs with IR spectroscopy. Primary amides as a case study. Crystengcomm.

[26] Saha S., Desiraju G.R. (2018). Acid center dot center dot center dot Amide Supramolecular Synthon in Cocrystals: From Spectroscopic Detection to Property Engineering. J. Am. Chem. Soc..

[27] Sarraguca M.C., Paisana M., Pinto J., Lopes J.A. (2016). Real-time monitoring of cocrystallization processes by solvent evaporation: A near infrared study. Eur. J. Pharm. Sci..

[28] Sarraguca M.C., Ribeiro P.R.S., Santos A.O., Silva M.C.D., Lopes J.A. (2014). A PAT approach for the on-line monitoring of pharmaceutical co-crystals formation with near infrared spectroscopy. Int. J. Pharm..

[29] Simone E., Saleemi A.N., Nagy Z.K. (2015). In Situ Monitoring of Polymorphic Transformations Using a Composite Sensor Array of Raman, NIR, and ATR-UV/vis Spectroscopy, FBRM, and PVM for an Intelligent Decision Support System. Org. Process Res. Dev..

[30] Simone E., Saleemi A.N., Nagy Z.K. (2014). Application of quantitative Raman spectroscopy for the monitoring of polymorphic transformation in crystallization processes using a good calibration practice procedure. Chem. Eng. Res. Des..

[31] Powell K.A., Croker D.M., Rielly C.D., Nagy Z.K. (2016). PAT-based design of agrochemical co-crystallization processes: A case-study for the selective crystallization of 1:1 and 3:2 co-crystals of p-toluenesulfonamideitriphenylphosphine oxide. Chem. Eng. Sci..

[32] Sarraguca M.C., Ribeiro P.R.S., Dos Santos A.O., Lopes J.A. (2015). Batch Statistical Process Monitoring Approach to a Cocrystallization Process. J. Pharm. Sci..

[33] Aitipamula S., Chow P.S., Tan R.B.H. (2014). Polymorphism in cocrystals: A review and assessment of its significance. Crystengcomm.

[34] Jolliffe I.T. (2002). Principal Component Analysis.

